# *Rickettsia parkeri* colonization in *Amblyomma maculatum*: the role of superoxide dismutases

**DOI:** 10.1186/s13071-016-1579-1

**Published:** 2016-05-20

**Authors:** Gary Crispell, Khemraj Budachetri, Shahid Karim

**Affiliations:** Department of Biological Sciences, University of Southern Mississippi, Hattiesburg, MS 39406 USA

**Keywords:** *Amblyomma maculatum*, *Rickettsia parkeri*, Tick, American boutonneuse fever, Superoxide dismutase, Reactive oxygen species, Selenoprotein, Lipid peroxidation, Bacterial load

## Abstract

**Background:**

The Gulf Coast tick (*Amblyomma maculatum*) is an arthropod vector of *Rickettsia parkeri*, the causative agent of American boutonneuse fever and an infectious agent of public health significance. In this study, we evaluated the biological significance of the superoxide dismutases (SODs) of *A. maculatum* in hematophagy and *R. parkeri* colonization within the tick host.

**Methods:**

An RNA interference approach was used to measure the functional roles of tick SODs (*Cu/Zn-SOD* and *Mn-SOD*) in *R. parkeri* colonization of the tick vector. Total microbial load, *R. parkeri* infection rate, and compensatory mechanisms by tick genes were examined using quantitative polymerase chain reaction (PCR) and quantitative reverse-transcriptase PCR assays. SOD enzymatic activity assays and malondialdehyde (MDA) lipid peroxidation were employed to determine the redox states in the tick tissues.

**Results:**

Knockdown of the *Cu/Zn-SOD* gene caused the upregulation of *Mn-SOD* in transcript levels. Single and dual knockdowns of the SOD genes caused an increase in MDA lipid peroxidation while SOD enzymatic activities did not show a significant change. *Mn-SOD* knockdown resulted in a substantial increase in the microbial load; however, *Cu/Zn-SOD* transcript depletion prompted an upsurge in the midgut bacterial load, and significantly decreased the bacterial load in salivary gland tissues. Additionally, *Cu/Zn-SOD* transcript silencing led to significantly fewer *R. parkeri* DNA copy numbers in both tick tissues (midguts and salivary glands).

**Conclusions:**

SOD enzymes play an important function in the regulation of bacterial communities associated with tick vectors and also in the defense mechanisms against the damage caused by reactive oxygen species within the tick. Knockdown experiments increased the levels of total oxidative stress in ticks, revealing the interplay between SOD isozymes that results in the transcriptional regulation of tick antioxidants. Moreover, the tick's *Cu/Zn-SOD* aids in the colonization of *R. parkeri* in tick tissues providing evidence of *A. maculatum's* vectorial success for a spotted fever group rickettsial pathogen.

**Electronic supplementary material:**

The online version of this article (doi:10.1186/s13071-016-1579-1) contains supplementary material, which is available to authorized users.

## Background

The Gulf Coast tick, *Amblyomma maculatum*, is a recently emerged arthropod that is posing an increasingly significant risk to public health [1]. This tick covers a geographical range encompassing the coastal areas of the Eastern Atlantic and the Gulf of Mexico within the United States. It is an arthropod vector of the spotted fever group rickettsial pathogen, *Rickettsia parkeri*, which causes American boutonneuse fever, a similar but milder form of Rocky Mountain spotted fever. Vector competence in ticks (pathogen acquisition, colonization, and transmission) is a multifactorial process involving several genes and numerous gene networks within the tick organs (midgut and salivary glands).

Tick blood meal digestion elevates the reactive oxygen species (ROS) level, which in turn can severely harm cellular components, thereby promoting apoptosis. Hydroxyl radicals, superoxide anions (O^2-^), and hydrogen peroxide (H_2_O_2_) are generated through ROS. The manganese superoxide dismutase (Mn-SOD) enzyme catalyzes a small percentage of O^2-^ to H_2_O_2_, as molecular oxygen is consecutively reduced to H_2_O by the electron transport chain complexes. Several other mitochondrial enzymes facilitate the additional reduction of H_2_O_2_ into H_2_O and molecular oxygen. Under normal circumstances, ROS partake in cell signaling by the intervention of select thiol residues in proteins, which have, among other capacities, a plausible role in regulating significant changes in transcriptional expression [2]. However, certain conditions can disturb the balance between the antioxidant capacity of the cell and an increase in the level of ROS. This condition, known as oxidative stress, causes permanent harm to macromolecules (proteins, lipids and DNA) and can eventually lead to cellular necrosis and cell death. Ticks must maintain homeostasis to survive, and they take blood meals of spectacular size, up to 100 times their unfed mass. Ticks must somehow avert the detrimental effects while promoting the beneficial aspects of ROS, and this suggests that there are specific factors for maintaining homeostasis within the tick itself and possibly at the tick–host interface. To achieve redox homeostasis in response to the detrimental effects of ROS, the tick antioxidant system acts through enzymes, such as catalase (*Cat*), superoxide dismutases (SODs), glutathione peroxide, glutathione-S-transferase, glutathione reductase (*GSHR*), selenoenzymes, and non-enzymatic molecules [3–6].

Localized ROS generation has been described in arthropods as the first line of defense against infectious agents [7, 8]. Tick-borne pathogens manipulate the gene expression of their host vector to ensure their survival, replication, and transmission to the mammalian host. Evidence of a well-organized tick antioxidant system lends support to the idea that tick-borne pathogens manipulate the system so that it is beneficial for their survival and colonization within the tick before inoculation into the mammalian host. While the role of ROS and antioxidants in the vectorial competence of ticks has not yet been explained, the redox balance and the growth and survival of *R. parkeri* in the tick host is an enigma. Hence, in this study, the biological implications of SODs in *R. parkeri* colonization within the *A. maculatum* vector are examined.

## Methods

### Ethics statement

All animal experiments were conducted in strict accordance with the recommendations in the Guide for the Care and Use of Laboratory Animals of the National Institutes of Health, USA. The protocol for tick blood feeding on sheep was approved by the Institutional Animal Care and Use Committee of the University of Southern Mississippi (protocol #15101501 and #15011402). All efforts were made to minimize animal suffering.

### Ticks and other animals

Gulf Coast ticks (*A. maculatum*) were maintained at the University of Southern Mississippi according to established methods [9]. Unfed adult ticks were obtained from Oklahoma State University’s Tick Rearing Facility (Stillwater, OK, USA). *Rickettsia parkeri*-infected *A. maculatum* ticks were maintained in the laboratory after collection from the field [10]. Adult ticks were kept at room temperature with approximately 90 % relative humidity under a photoperiod of 14 h light/10 h dark before infestation on sheep. Ticks were blood-fed on sheep and were either allowed to feed to repletion or were removed at 3–10 days after attachment, depending on the experimental protocol. Adult ticks were fed on sheep and immature ticks were fed on hamsters (specifically used for this study), and the animal studies were performed in accordance with protocols approved by the Institutional Animal Care and Use Committee (IACUC) at the University of Southern Mississippi.

### Bioinformatics analyses

The coding sequences of SODs for *A. maculatum* genes used in this study were obtained from an *A. maculatum* sialotranscriptome study [3]. Nucleotide sequences were conceptually translated and initially aligned using ClustalX2 [11, 12] and graphically presented using Jalview [13]. Phylogenetic relationships were inferred by MEGA 6 [14] using maximum likelihood method using JTT matrix-based model [15].

### Tick midgut and salivary gland preparation

The unfed and partially blood-fed female adult ticks were dissected within 4 h of removal from the sheep. The remarkable contribution of midgut and salivary gland tissues in tick’s hematophagy, and potential role of these tissues in pathogen colonization, propagation, and saliva-assisted transmission to the vertebrate host led us to focus on these tissues in this study [16]. The tick midgut and salivary gland tissues were dissected in ice-cold M-199 buffer as described by Morgan et al. [17, 18]. After removal, the midguts and salivary glands were washed gently in the same ice-cold buffer. The tick tissues were either stored immediately after dissection in RNAlater (Invitrogen, Carlsbad, CA, USA) prior to mRNA extraction, or in protein extraction buffer.

### RNA preparation, cDNA synthesis, and quantitative reverse-transcriptase (qRT)-PCR

Extraction of total RNA, cDNA synthesis, and qRT-PCR were conducted as previously described [19]. Briefly, the tick midguts and salivary gland tissues stored in RNAlater were used for total RNA extraction using an Illustra™ RNAspin Mini Isolation kit (GE Healthcare, Piscataway, NJ, USA) according to the manufacturer’s instructions. The RNA concentration was determined using a Nanodrop spectrophotometer (Thermo Fisher Scientific, Wilmington, DE, USA). Total RNA (1 μg) was reverse transcribed into cDNA using iScript cDNA synthesis kit (Bio-Rad Inc., Hercules, CA, USA) according to the manufacturer’s instructions . The gene-specific primer sequences designed to amplify specific cDNA fragments from *A. maculatum* tissues are listed in the Table. Transcriptional gene expression of the SODs in uninfected (naïve) ticks was normalized against the *β-actin* gene, while glyceraldehyde 3-phosphate dehydrogenase (*GAPDH*) was used to normalize gene expression in *R. parkeri*-infected tick tissues. The selection of *β-actin* and *GAPDH* reference genes was based on their stable gene expression levels in uninfected and rickettsial-infected ticks [19]. All genes used in this study were first amplified using gene-specific primers (Table 1), and their sequences were confirmed by sequencing prior to dsRNA synthesis or gene expression studies. First-strand cDNA was used to measure mRNA levels using qRT-PCR. SYBR Green qPCR Master Mix (Bio-Rad Inc., Hercules, CA, USA), 25 ng of cDNA, and 150 nM of gene-specific primers were used in each reaction mixture [6]. The qRT-PCR mixtures were subjected to 10 min at 95 °C, followed by 35 cycles of 15 s at 95 °C, 30 s at 60 °C, and 30 s at 72 °C using the CFX96 Real Time System (Bio-Rad Inc.).

**Table 1:** Gene-specific quantitative reverse-transcriptase PCR primers used in the experiments

Gene	Accession number	Forward primer 5′-3′	Reverse primer 5′-3′	Size (bp)
*TrxR*	JO843723	TGTGACTACACCAACGTGCCTACA	AGTAGCCTGCATCCGTTCCTCTTT	175
*Catalase*	JO843741	AAAGGACGTCGACATGTTCTGGGA	ACTTGCAGTAGACTGCCTCGTTGT	173
*GSHR*	JO844062	ACCTGACCAAGAGCAACGTTGAGA	ATCGCTTGTGATGCCAAACTCTGC	170
*MnSOD* (*SOD3*)	JO843979	GCATCTACTGGACAAACCTCTC	GCAGACATCAGGCCTTTGA	115
*Cu/ZnSOD* (*SOD1*)	JO844140	GGAACCGAAGACAGCAAGAA	GAGAAGAGGCCGATGACAAA	143
*Duox*	N/A	ATGACGCACAGCCTGTATATT	TGTCCAGAGTGAAGACGATTG	123
*16S rRNA*	N/A	AGAGTTTGATCCTGGCTCAG	CATGCTGCCTCCCGTAGGAGT	N/A
*Actin*	JO842238	TGGCTCCTTCCACCATGAAGATCA	TAGAAGCACTTGCGGTGCACAATG	169
*GAPDH*	JO842341	CACCCATCACAAACATGGGTGCAT	TTTCAGGAAATGAAGCCTGCCAGC	175
*OmpB*	AF123717	CAAATGTTGCAGTTCCTCTAAATG	AAAACAAACCGTTAAAACTACCG	96
Probe: 6-FAM-CGCGAAATTAATACCCTTATGAGCAGCAGTCGCG-BHQ-1				
*SelM*	JO842653	ATGATACCTGAATGGCCATCCGCA	TGATCGCGGGTCATCTTCTCCAAA	171
*eEFSec (SEF)*	KC989559	TGGCTCCAGAAATGCTGCTCATTG	ACGCCTTTGCGACTCTTCTCCTTA	157
*SelS*	JO842687	AGAACAAGTGCACCACAACAGCAG	ATTTCTTGCATCCTTCGACGTGCC	107
*SelN*	KC989560	TTAGTTTGGACACTGTGGACGGGT	AGGCTTCTCTAACAACGGCACTCA	150
*SelK*	JO843326	AGTTCCAGCAGGTCATCAGTGTCA	TCCAGGAATAGGGCAGTCCATTGT	132
*Salp25D*	JO843645	TGCCGCGCTGTCTTTATTATTGGC	AGTTGCACGGAGAACCTCATCGAA	102

### Double-stranded RNA (dsRNA) synthesis, tick injections, and hematophagy

Synthesis of dsRNA for SOD analysis and tick manipulations was performed according to the methods described previously [20, 21]. Briefly, PCR product, purified using a QIAquick PCR purification kit (Qiagen, Valencia, CA, USA), was used in a secondary PCR using the same primers with SOD-specific sequences (Table 1), but with the addition of flanking T7 sequences that allow for the binding of reverse transcriptase and the generation of dsRNA. After confirming the T7-flanked SOD gene sequence, the secondary PCR product was reverse transcribed into RNA using a T7 Quick High Yield RNA synthesis kit (New England Biolabs, Ipswich, MA, USA) by incubating the PCR product with T7 polymerase overnight at 37 °C. The resulting dsRNA was purified by ethanol precipitation and its concentration measured spectrophotometrically using a Nanodrop device (Thermo Fisher Scientific). The product was visualized by gel electrophoresis using a 2 % agarose gel. The dsRNAs (dsMn-SOD and dsCu/Zn-SOD) were diluted to working concentrations of 1 μg/μl. The same protocol was used to synthesize dsRNA-LacZ to be used as a dsRNA control. Forty-five unfed adult female ticks (uninfected or *R. parkeri*-infected) were microinjected with 1 μl of dsRNA-SOD or dsLacZ using a 27-gauge needle, after which they were kept overnight at 37 °C to alleviate needle trauma and promote survival. The surviving ticks were used to infest a sheep and allowed to blood feed in the presence of male ticks. For sample collection, 10 partially fed experimental control ticks were removed on days 5 and 7 post-tick infestation, and the remaining ticks were allowed to remain attached and blood feed until repletion. The feeding success of the individual ticks was evaluated by recording their attachment duration, repletion weight, and the ability to oviposition [22]. Partially fed ticks removed from the dsLacZ or dsSOD groups were dissected to obtain their midguts and salivary gland tissues.

### Quantification of total bacterial load

The bacterial load in each tick tissue was estimated as described previously [6, 23]. Briefly, 25 ng of cDNA from the tick tissues, 200 μM *16sRNA* gene primers, and 2× iTaq Universal SYBR green mix (Bio-Rad Inc.) in a 25-μl volume reaction was used with the following thermocycler parameters: 94 °C for 5 min followed by 35 cycles at 94 °C for 30 s, 60 °C for 30 s and 72 °C for 30 s. Standard curves were used to estimate the copy numbers of each gene. The bacterial copy numbers were normalized against *A. maculatum* actin copy numbers in the uninfected ticks and *GAPDH* in the *R. parkeri*-infected ticks. All samples were run in triplicate.

### Quantification of *R. parkeri* copy numbers in tick tissues

The level of infection with *R. parkeri* within the tick tissues (midguts and salivary glands) was quantified using a probe-based qPCR method described previously [10]. Briefly, 0.4 μM of probe, 0.7 μM of each forward and reverse primer (Rpa129F, Rpa224R) (Table 1) and 8 mM of MgSO_4_ reaction mixture was subjected to one cycle each of 50 °C for 2 min and 95 °C for 2 min, then 45 cycles of 95 °C for 15 s and 60 °C for 30 s. A standard curve was used to calculate the *R. parkeri* copy numbers in the tick samples. All samples were run in triplicate.

### Quantification of total oxidative stress levels

A malondialdehyde (MDA) lipid peroxidation assay was used to quantify the total oxidative stress levels in the tick tissues. Degradation of lipids as a result of oxidative damage was estimated by quantification of MDA through the use of a Lipid Peroxidation MDA Assay Kit (Sigma-Aldrich, St. Louis, MO, USA) following the manufacturer provided protocol. Twenty milligrams of midgut tissue from individual ticks was filtered through a 0.2 μm filter before the assay was performed according to the manufacturer’s instructions.

### Quantification of SOD activity

Superoxide enzymatic activity was quantified in individual midgut and salivary gland tissues using the Superoxide Dismutase Assay Kit (Cayman Chemical Co, Ann Arbor, MI, USA). The tissues were processed according to the manufacturer’s instructions.

### Data analysis

All data are expressed as mean ± SEM unless otherwise stated. Statistical significance between the two experimental groups or their respective controls was determined by the *t*-test). Comparative differences amongst the multiple experimental groups were determined by analysis of variance with statistically significant *P*-values of < 0.05 (Graphpad Prism 6.05, La Jolla, CA, USA). Transcriptional expression levels were determined using Bio-Rad software (Bio-Rad CFX MANAGER v.3.1), and the expression values were considered significant if the *P*-value was 0.05 when compared with the control.

## Results

### Bioinformatic analyses

In this study, the functional role of *A. maculatum* superoxide dismustases (SODs) in hematophagy, and bacterial colonization of endosymbionts and pathogenic microbe *R. parkeri* was determined. Two gene sequences of tick superoxide dismutases cytosolic with signal peptide (*Cu/ZnSOD*) and intramitrocondrial scavenger (*Mn-SOD*) were selected to analyze for the presence of secretory signal peptide using SignalP [24] server (http://www.cbs.dtu.dk/services/SignalP/). The amino acid residues deduced from the transcripts of two SODs identified earlier [3] were translated and visualized using Jalview and observed six histidine residues in each sequences showing metal-binding sites (Additional file 1: Figure S2A, B). The four histidine imidazoles are coordinated to Cu(II) while two other and including one common histidine imadazoles binds to Zn(II) metal in Cu/Zn-SOD dismutase [25]. The Mn-SOD localized in mitochondria in eukaryotes, and found in cytoplasm in prokaryotes shares common structural properties with Fe-SOD [26]. The phylogenetic tree was built in MEGA software version 6 [14] to provide evolutionary significance between mitochondrial and cytoplasmic superoxide dismutases (Additional file 1: Figure S1). Phylogenetically Mn-SOD and Cu/Zn-SOD fit in two different clades, sharing common node with corresponding SODs from another *Amblyomma* ticks (Additional file 1: Figure S1).

**Fig. 1:**
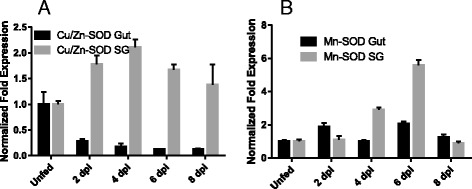
Time-dependent *Cu/Zn-SOD* and *Mn-SOD* transcriptional expression levels in uninfected (naïve) tick midgut and salivary gland tissues during the blood meal. The relative transcript levels of the SODs during the blood meal were measured by comparing the SOD expression levels in the midgut (Gut) and salivary gland (SG) tissues of the unfed-stage ticks. The changes in transcriptional activity of (**a**) *Cu/Zn-SOD* and (**b**) *Mn-SOD* in the *A. maculatum* midgut and salivary gland tissues were normalized to the unfed developmental stage using *β-actin* as a reference gene

### Time-dependent transcriptional gene expression analysis

The time-dependent transcriptional expression of the *Cu/Zn-SOD* and *Mn-SOD* genes was examined in unfed and partially blood-fed tick tissues (midguts and salivary glands) (Fig. 1). The time-dependent expression of the *Cu/Zn-SOD* gene in the tick midgut tissues showed its highest transcript level during the unfed phase followed by significant depletion after attachment, and the transcript level remained down-regulated as the tick blood feeding switched from day 2 to day 8 (Fig. 1a). Interestingly, the transcript level of the *Cu/Zn-SOD* gene in the tick salivary glands was up-regulated during the slow and fasting feeding phases of tick blood feeding, signifying a potential functional role in tick hematophagy (Fig. 1a). By contrast, the transcript level of the *Mn-SOD* gene in the tick midgut tissues upon blood feeding was up-regulated and remained 2-fold higher than in the unfed tissues in the 2- and 6 day-fed ticks (Fig. 1b); however, the salivary glands showed a 2- to 6-fold increase in the transcript level on days 4 and 6 post-attachment to the vertebrate host (Fig. 1b). Surprisingly, the transcript level of *Mn-SOD* decreased in the fast-feeding phase (8 days post-infestation), implying compensation by *Cu/Zn-SOD* or a reduced biological need in the cell.

### Impact of single and multiple SOD knockdowns in uninfected ticks

**Fig. 2:**
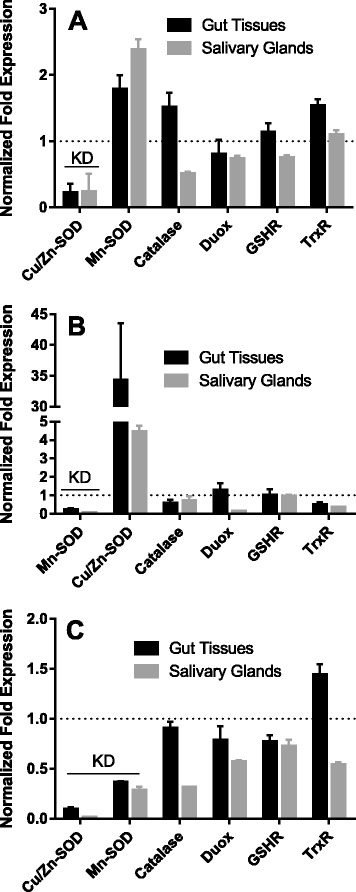
Knockdown of tick SODs in naïve (uninfected) *A. maculatum* and the effect on tick antioxidants. **a** *Cu/Zn-SOD*, **b** *Mn-SOD*, **c** *Cu/Zn-SOD + Mn-SOD* dual knockdowns. Gene expression in naïve ticks was normalized against tick *β-actin*, used as the reference gene. The transcript levels of the target genes in *dsLacZ* injected tick tissues were made equal to 1 and represented by a dashed line in the graphs. KD; Knockdowns

**Fig. 3:**
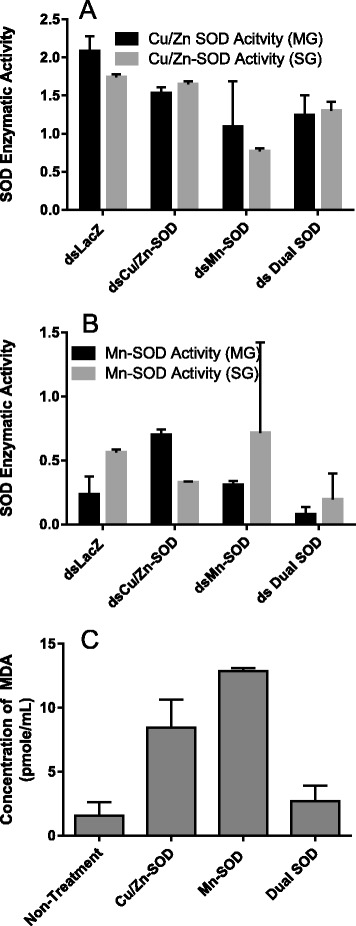
Quantification of the SOD activity and total oxidative stress levels in superoxide dismutase-silenced naïve tick midguts. **a** Cytosolic and **b** mitochondrial SOD enzymatic activities in tick midgut and salivary gland tissues were estimated using Superoxide Dismutase Assay Kit (Cayman Chemical Co, Ann Arbor, MI, USA). The tissue cytosolic fraction was used for determining the enzymatic activity of Cu/Zn-SOD, and mitochondrial fraction was reconstituted to assess Mn-SOD activity. **c** A Lipid Peroxidation (MDA) Assay Kit (Sigma-Aldrich, St. Louis, MO, USA) was used for estimating total oxidative stress in the tick midgut tissues. The MDA-TBA adduct was spectrometrically estimated at 532 nm in each control and knockdown tick midgut

**Fig. 4:**
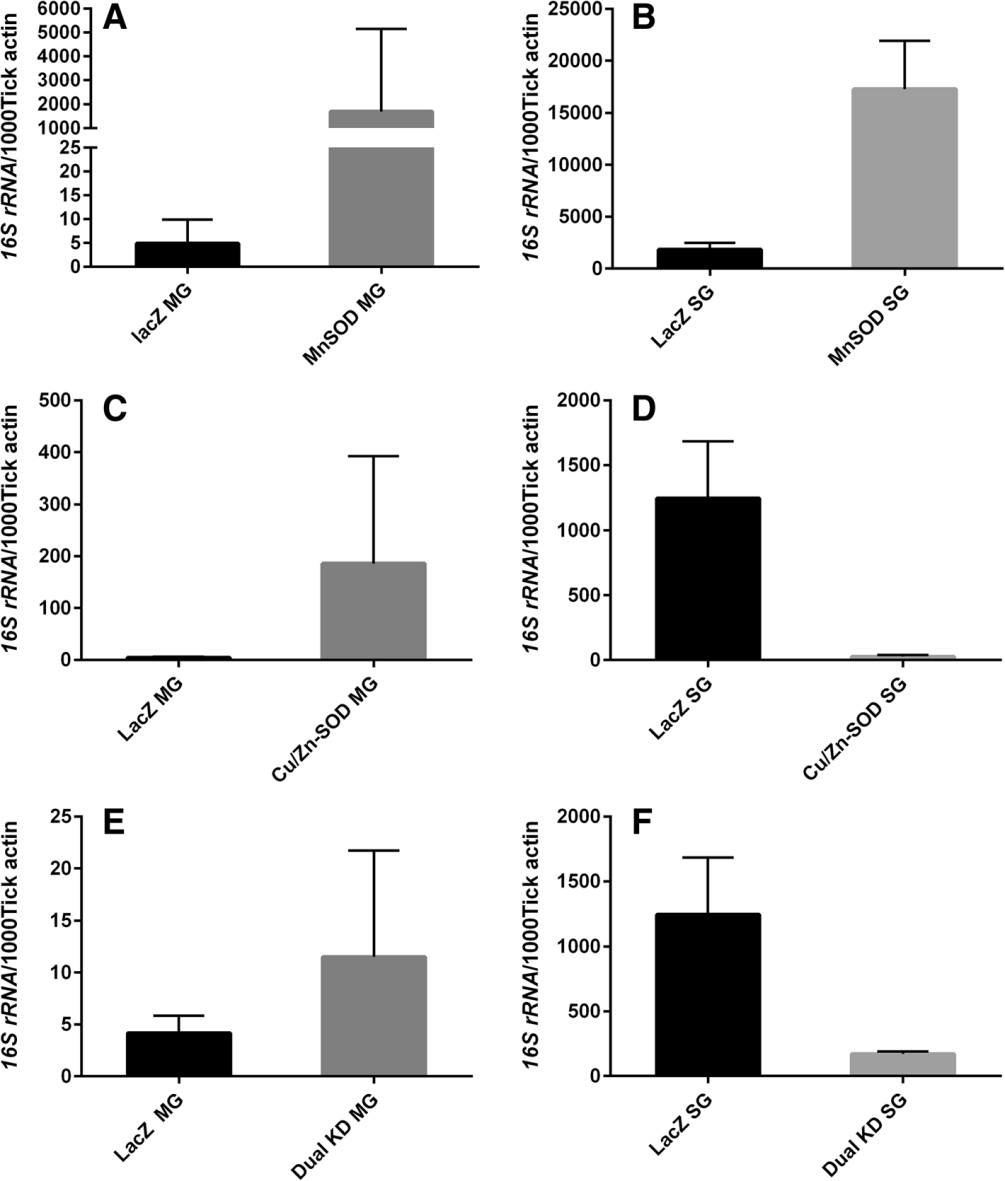
Impact of SOD knockdown on microbiota load in naïve tick tissues. The bacterial load in the tick tissues (measured in copy numbers) was calculated by qRT-PCR using the bacterial *16 s rRNA* gene and was normalized against the tick *β*-*actin* gene (copy number) in tick midguts and salivary glands each knockdown; *Mn-SOD* (**a**, **b**); *Cu/Zn-SOD* (**c**, **d**); and Dual SODs knockdown (**e**, **f**)

Transcriptional gene expression: To investigate the functional role(s) of the secreted extracellular and mitochondrial SOD enzymes, genes were disrupted using RNA interference, and the effects of the single and dual knockdowns upon the transcriptional gene expression of *Cat*, *dual oxidase* (*Duox*), *GSHR* and thioredoxin reductase, as well as oxidative stress, bacterial load, and enzymatic activity was investigated (Figs. 2, 3 and 4). A *Cu/Zn-SOD* gene silencing efficiency of 77 and 76 % was achieved in 5 dpi tick midgut and salivary gland tissues respectively (Fig. 2a). On the contrary, *Mn-SOD* transcript silencing efficiency of 76 and 94 % was obtained in tick midgut and salivary tissues respectively (Fig. 2b). Intriguingly, simultaneous gene silencing of *Cu/Zn-SOD* and *Mn-SOD* showed 90 and 98 % silencing efficiency of *Cu/Zn-SOD* verses 63 and 71 % of *Mn-SOD* in midgut and salivary gland tissues respectively (Fig. 2c). Regardless of the single (*Cu/Zn-SOD* or *Mn-SOD*) and dual (*Cu/Zn-SOD* and *Mn-SOD*) knockdowns, there were no significant changes in tick behavior or phenotype in terms of the tick engorgement weight, the attachment duration (Additional file 1: Figure S3). Surprisingly, knockdown of tick SODs did not translate into the depletion of SOD enzymatic activity in midgut tissues but salivary glands showed a decrease in *Cu/Zn-SOD* and dual knockdown tissues, dsMn-SOD (*P* = 0.002), and dual SOD injected (*P* = 0.036) (one-way ANOVA using multiple comparison with Tukey test) (Fig. 3a, b). The lack of correlation between Cu/Zn-SOD level may be due to the residual amount of proteins already present in the tissues. *Cu/Zn-SOD* silencing in the partially blood-fed tick tissues up-regulated *Mn-SOD* gene expression; however, *Cat*, *Duox*, and *GSHR* gene expression in the salivary glands remained unchanged (Fig. 2a). By contrast, *Mn-SOD* silencing in uninfected (naïve) ticks showed a 4–35-fold increase in *Cu/Zn-SOD* expression and, intriguingly, *Duox* expression in the salivary glands was significantly down-regulated (*P* < 0.05), suggesting an interplay between *Duox* and *Mn-SOD* in the salivary glands (Fig. 2b). Simultaneous silencing of *Cu/Zn-SOD* and *Mn-SOD* in naïve ticks only impacted the expression of selected genes in the salivary gland tissues (Fig. 2c), suggesting the tissue-specific expression of antioxidant genes during blood feeding.

Oxidative stress levels and SOD enzymatic activity: The cytosolic and mitochondrial SOD enzymatic activities were determined in the uninfected single- and simultaneously gene-silenced tick midgut and salivary gland tissues. Knockdown of *Cu/Zn-SOD* resulted in slightly decreased enzyme activity, while *Mn-SOD* silencing lowered the activity in the midgut and salivary gland tissue fractions compared with the control (Fig. 3a). Simultaneous silencing of *Cu/Zn-SOD* and *Mn-SOD* produced lower *Cu/Zn-SOD* activity in the midgut and salivary gland tissues; while the salivary glands experienced a slight decrease in Mn-SOD activity as compared with the irrelevant dsRNA-treated tick tissues (Fig. 3b). Additionally, the level of oxidative stress, as quantified by MDA, showed a 5–6-fold increase in oxidative stress upon knockdown of *Cu/Zn-SOD* (*t* = 2.847, *P* = 0.0465) and *Mn-SOD* (*t* = 10, *P* = 0.0004) in the midgut tissues; however, simultaneous knockdown of the expression of both genes produced only a 2-fold increase (*t* = 0.6904, *P* = 0.529) in the oxidative stress level (Fig. 3c). Quantification of the oxidative stress level in the salivary gland tissues was hampered by the insufficiently high protein concentration needed for this assay.

Native microbial load: To further understand the role of SODs in the native microbial load within the tick tissues, we calculated the overall bacterial load by quantifying the *16S rRNA* gene. We observed that when *Mn-SOD* was knocked down in the uninfected tick tissues the total bacterial load in the midgut and salivary gland tissues increased (*F*_(3, 36)_ = 9.636, *P* < 0.0001) (Fig. 4a and b). By contrast*, Cu/Zn-SOD* gene silencing significantly depleted the colonization of microbiota in the tick salivary glands (*t* = 2.794, *P* = 0.0190) (Fig. 4d), suggesting a hypothetical role for this gene in the colonization of microbes in tick salivary glands before saliva-assisted transmission to the host (Fig. 4b). Intriguingly, *Cu/Zn-SOD* gene silencing resulted in no significant difference in the microbial load in the tick midgut tissues (*t* = 2.151, *P* = 0.0570) (Fig. 4c). The bacterial load in tick tissues was significantly altered in dual knockdown of SODs in tick tissues (*F*_(3, 20)_ =7.493, *P* = 0.0015) (Fig. 4e, f).

### Impact of *Cu/Zn-SOD* knockdown in *R. parkeri*-infected ticks

**Fig. 5:**
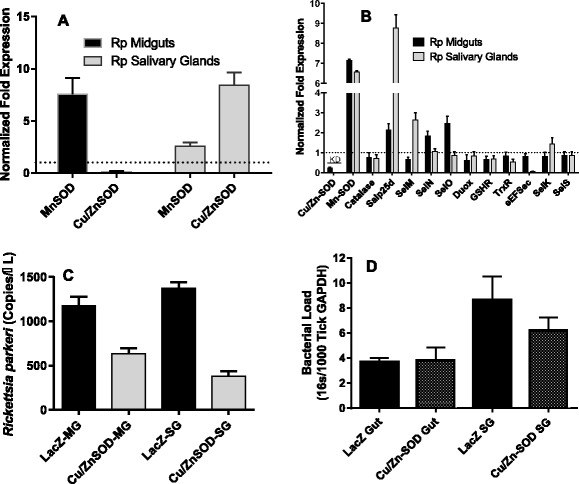
Knockdown of *Cu/Zn-SOD* in *R. parkeri*-infected *A. maculatum*. **a** Tissue-specific SOD transcriptional gene expression in uninfected and *R. parkeri*-infected partially blood-fed female adults tissues. *A. maculatum GAPDH* was used as a reference gene to normalize the gene expression data. Target gene expression in uninfected tick tissues was adjusted to 1. **b** Transcriptional gene expression of selected antioxidant genes in *Cu/Zn-SOD*-silenced partially fed tick tissues. The transcript level of each target gene in the tissues injected with control LacZ-dsRNA was set to 1.0 as a reference point. Expression was normalized against the tick *GAPDH* gene. **c** *R. parkeri* was quantified using a real-time PCR assay designed for specific detection of the *R. parkeri ompB* gene in *A. maculatum* midgut and salivary gland in dsLacZ and *dsCu/Zn-S*OD injected tick tissues, and (**d**), the bacterial load estimation in *R. parkeri*-infected ticks knocked down with *Cu/ZnSOD*. Tick *GAPDH* was used as a reference gene for estimating the total bacterial load in *R. parkeri*-infected ticks. KD; Knockdowns

SOD expression was assessed in the tissues (midgut and salivary glands) of uninfected and *R. parkeri*-infected partially blood-fed adult ticks (Fig. 5a). Interestingly, the *Mn-SOD* transcript level was up-regulated 8-fold while *Cu/Zn-SOD* was down-regulated in the *R. parkeri*-infected tick midgut tissues (Fig. 5a). *Rickettsia parkeri* infection in the partially blood-fed salivary glands caused up-regulation of the expression of both SODs (2–8-fold, *P* < 0.05), suggesting their potential role in pathogen colonization within the tick host (Fig. 5a). To further understand the proposed role of *Cu/Zn-SOD* in *R. parkeri* colonization in *A. maculatum*, *Cu/Zn-SOD* was silenced using RNAi, and its effect on the native microbial load and *R. parkeri* colonization was examined (Fig. 5c, d). As anticipated, the *Cu/Zn-SOD* transcript in the knockdown showed 77 and 99 % silencing efficiency in midgut and salivary gland tissues respectively in 5 dpi ticks (Fig. 5b) (*P* < 0.05). Predictably, the transcriptional expression of *Mn-SOD* exhibited a 6.5–7-fold up-regulation upon silencing of *Cu/Zn-SOD* in the partially blood-fed infected tissues (Fig. 5b). The transcript level of selected antioxidant genes (*Cat*, *Duox*, *GSHR*, and *TrxR*) decreased slightly in the *Cu/Zn-SOD*-silenced *R. parkeri*-infected tick tissues. Intriguingly, *Cu/Zn-SOD* silencing up-regulated the transcript level of selenoprotein O, *Mn-SOD*, and glutathione peroxidase (*Salp25D*) in the tick midguts, while *Mn-SOD* and *Salp25D* expression in the salivary gland tissues increased by 6–8-fold, indicating the existence of a compensatory mechanism (Fig. 5b). Interestingly, quantification of *R. parkeri* in the gene-silenced partially blood-fed tick tissues revealed a significant decrease in the rickettsial copy numbers in midguts (*t* = 7.5, *P* < 0.0001) and in salivary gland tissues (*t* = 8.419, *P* < 0.0001) (Fig. 5c), suggesting a potential role for *Cu/Zn-SOD* in *R. parkeri* colonization within the tick host. Surprisingly, the total bacterial load in *Cu/Zn-SOD* knockdown midgut (*t* = 0.5079, *P* = 0.6225) and salivary gland tissues (*t* = 0.03569, *P* = 0.09722) remained unchanged (Fig. 5d).

## Discussion

Ixodid ticks are arthropods dependent on hematophagy for transition to each life stage and for maintaining their reproductive fitness. Hematophagy in ixodid tick is a phase-dependent process, starting with host attachment and establishment of the feeding site, followed by slow feeding and transition into fast feeding until repletion. At this point in the feeding process, the tick can weigh over 100 times its pre-engorgement weight as a result of the imbibed blood meal. The entire process of tick feeding is dependent on the tick’s secreted salivary components that mechanistically facilitate successful transition of each feeding phase over the course of multiple days to weeks. Tick saliva, as shown by sialotranscriptome analysis, is composed of ~5000 putative secreted salivary proteins containing dozens of protein families [3, 27], many of which exhibit pharmacological properties capable of disarming the host’s attempts to establish hemostasis or to mount a specific immune response and, ultimately, enhancing the tick’s ability to blood-feed. At the same time, ticks face elevated oxidative stress both on and off the mammalian host and they must neutralize the harmful effect. The ability of ticks to counterbalance nutritional stress (starvation) and blood feeding-related stress (heme digestion) indicates the likely development of a proactive detoxification system. Complete studies of the antioxidant systems of blood-sucking arthropods are presently unavailable; nonetheless the scientific literature shows that ticks have an extensive arsenal of antioxidants and selenoproteins that allow them to survive extreme variations in redox homeostasis, with prolonged periods of starvation, the digestion of huge blood meals, and exposure to blood-related products [4–6, 20, 27]. Hence, an insight into the tick’s antioxidant repertoire should offer a glimpse of its defenses against elevated levels of oxidative stress. It should also broaden our understanding of tick–pathogen interactions. Although, *A. maculatum* is a recognized arthropod vector of *R. parkeri*, this vector-pathogen pair remains the most poorly studied vector-borne disease. Here, we investigated the functional role of two tick SODs (extracellular and mitochondrial) in tick hematophagy and pathogen colonization within the arthropod vector. Bioinformatic analysis of the *Cu/Zn-SOD* (cytosolic) and *Mn-SOD* proteins showed amino acid sequence similarity with arthropod and vertebrate proteins (Additional file 1: Figures S1 and S2).

The two *A. maculatum* SODs we characterized showed quite different expression patterns. The transcriptional activity *of Cu/Zn-SOD* was 2-fold up-regulated throughout the blood meal in the salivary tissues, whereas it declined in the midgut tissues upon blood meal consumption (Fig. 1a). *Mn-SOD* expression was up-regulated 3–6-fold in the salivary glands on days 4 and 6; however, the midgut maintained a steady expression level (Fig. 1b). Furthermore, while we obtained both single and dual gene transcriptional knockdowns, significant differences in the tick phenotype (tick engorgement weight, attachment duration, egg mass, egg conversion ratio, and hatchability) (Additional file 1: Figure S3) were lacking, a result that is similar to previous studies reporting the existence of a strong compensatory mechanism in the tick host for detoxifying superoxide and H_2_O_2_ [20]. The tick salivary glands undergo tremendous biochemical and physiological changes as soon as a tick attaches itself to the host, followed by engorgement and repletion [28]. In ticks, breakdown of the huge blood meal generates toxic levels of heme leading to elevated ROS production. The gut bacterial community is strongly shaped by the ROS levels upon a blood meal, resulting in a decrease in diversity and an increase in Enterobacteriaceae [6]. The establishment of vector competence (pathogen survival, colonization, and transmission) is highly specific: *R. parkeri* can only be vectored by *A. maculatum*, *Ehrlichia chaffeensis* by *Amblyomma americanum*, and *Borrelia burgdorferi* by *Ixodes scapularis*. It is postulated that these vector-borne disease agents have developed an antioxidant capacity that aids them to survive and multiply by balancing oxidative tissue-specific homeostasis. Tick-borne pathogens are vulnerable to a high level of oxidative stress; consequently, disrupting the redox metabolism offers a promising approach for the prevention of tick-borne disease agents and for disturbing tick microbiota. Our recent work highlighted the biological implication of thioredoxin reductase (TrxR, a selenoprotein) in preserving the natural microbiota of ticks [6], and a link between selenocysteine elongation factor and *R. parkeri* survival in the midgut tissues [4]. It has been shown that elimination of ROS is required to conserve fertility in *Anophele*s, *Drosophila*, *A. maculatum*, and mammals [20, 29–31]. To neutralize the damaging effects of ROS and attain homeostasis, tick SODs and Cat enzymes act together to catalyze the conversion of superoxide and H_2_O_2_. Superoxide and hydrogen peroxide facilitate the generation of the hydroxyl radical, the most reactive oxygen free radical, in the presence of iron metal [32, 33]. Interestingly, lipid peroxidation assays showed higher total oxidative stress levels for single knockdown tick midguts, while dual knockdown revealed only a slight change in the oxidative stress level, suggesting the presence of a strong compensatory mechanism, as has been reported previously (Fig. 3c) [20]. Estimation of the total oxidative stress levels in individual tick salivary glands was not successful because of the low protein concentrations required for this assay. Intriguingly, gene knockdown of *Cu/Zn-SOD* or *Mn-SOD* significantly increased the total bacterial load in the midgut tissues (Fig. 4a, c), whereas *Cu/Zn-SOD* knockdown in the salivary glands significantly reduced the total bacterial load (Fig. 4d). The total bacterial load in the dual SOD knockdown remained at a decreased level in both tissues. The interplay between ROS levels and survival and the colonization of intracellular bacteria has not been elucidated. Our results indicate that the microbes associated with ticks are dependent on maintaining redox homeostasis while also producing ROS within the tick cells, and they use the ROS as a signaling molecule to regulate the bacterial density [34]. Another possible interpretation is that *Cu/Zn-SOD* gene knockdown also affected the enzymatic activity of Mn-SOD (Fig. 3a, b), and the increased generation of superoxide in the salivary glands significantly reduced the total bacterial load. Intriguingly, redox homeostasis is routinely attained by counterbalancing ROS and antioxidants by regulating oxidative stress-induced genes [35], including Cat. The transcriptional expression of *Cat* was up-regulated slightly in the midgut tissues upon *Cu/Zn-SOD* knockdown; however, a compensatory role for *Cat* in increasing the bacterial load cannot be ruled out (Figs. 2a and 4). SOD facilitates the breakdown of O^2−^ to H_2_O_2_ and molecular oxygen. *Mn-SOD* (*SOD2*) from *Drosophila* mutants show an increased herbicide (Paraquat) sensitivity, elevated endogenous oxidative stress, and reduced longevity [36]. A significant decrease in the total bacterial load upon *Cu/Zn-SOD* knockdown led us to a follow-up experiment to study the functional role of *Cu/Zn-SOD* gene depletion on *R. parkeri* colonization within the tick host.

ROS provide protection against a variety of pathogens by eliciting immune responses in organisms as diverse as mammals and arthropods, including tick responses against *R. parkeri* [4]. Fascinatingly, the interactions between *A. maculatum* and *R. parkeri* determine the ability of these ticks to become colonized by and transmit rickettsial agents. In tick-transmitted infections, the infectious agents manipulate gene expression of the vector host to ensure their colonization and onward transmission to the mammalian host. In the current study, *Cu/Zn-SOD* and *Mn-SOD* transcript levels in the *R. parkeri*-infected ticks showed differential gene expression. Interesting, the transcript level of *Mn-SOD* significantly upregulated (*P* < 0.05) in *R. parkeri-*infected partially blood fed tick tissues and *Cu/Zn-SOD* transcript level increased only in salivary glands (*P* < 0.05) and remained down-regulated in gut tissues (Fig. 5a). It suggests that tick protects herself from the elevated level of super-oxides generated and released upon rickettsial infection by regulating the expression of superoxide dismutase [37] (Fig. 5a). Interestingly, downregulation of *Cu/Zn-SOD* level in *R. parkeri* infected midguts co-related with alleviated *Cu/Zn-SOD* transcript abundance in partially blood-fed tissues (Fig. 1a). As expected, the *Cu/Zn-SOD* knockdown up-regulated *Mn-SOD* (~6.5-fold) expression in the midgut and salivary glands. Surprisingly, the expression levels of *Salp25D* and *SelM* were 9-fold and 3-fold up-regulated, respectively. It is established that *A. maculatum eEFSec*, *I. scapularis* glutathione peroxidase (*Salp25D*), and *Dermacentor variabilis SelM* offer a survival advantage to *R. parkeri*, *B. burgdorferi,* and *Anaplasma marginale* [4, 38, 39].

## Conclusions

This is the first study to show that the silencing of *Cu/Zn-SOD * decreases the level of *R. parkeri* colonization in tick tissues. The *A. maculatum* antioxidants, *Mn-SOD* and *Cu/Zn-SOD* play a role in maintaining redox homeostasis within the tick and facilitate the colonization of human pathogenic bacteria, *R. parkeri*. Our findings provide a strong foundation for future research to elucidate the molecular determinants of vector competence. The relationship between ROS, vector competence, and *R. parkeri*-induced antioxidants is undergoing further investigation in our laboratory.

## Additional files

Additional file 1:
**Figure S1.**  Evolutionary relationships of taxa based on the SOD amino acid sequence using maximum likelihood method. The evolutionary history was inferred by using the Maximum Likelihood method based on the JTT matrix-based model [15]. The tree is drawn to scale, with the branch lengths measured by the number of substitutions per site. Evolutionary analyses were conducted using MEGA6 software [14]. The sequences were obtained from *Amblyomma maculatum*, *Amblyomma variegatum*, *Ixodes scapularis*, *Anopheles gambiae*, *Mus musculus*, *Sus scrofa*, and *Homo sapiens*. GenBank accession numbers followed by species names are shown in the tree. **Figure S2A.** Multiple sequence alignments of Cu/Zn-SOD amino acid sequences from different taxa. Regions outlined by red boxes indicate metal-binding sites that are conserved between all of the listed species. The sequences for Cu/Zn-SODs were obtained from *Drosophila melanogaster*, *Apis mellifera*, *Amblyomma maculatum*, Thermostable mutant of Human Cu/Zn SOD, *Mus musculus*, *Equus caballus*, *Danio rerio*, *Anopheles gambiae* and *Culex quinquefasciatus*. **Figure S2B.** Multiple sequence alignments of Mn-SOD amino acid sequences from different taxa. Regions outlined by red boxes indicate metal-binding sites that are conserved between all the listed species. The sequences were obtained from *Haliotis discus*, *Lottia gigantea*, *Perinereis nuntia*, *Stegastes partitus*, *Takifugu rubripes*, *Struthio camelus*, *Tauraco erythrolophus*, *Thamnophis elegans*, *Sus scrofa*, *Macaca mulatta*, *Mus musculus*, *Xenopus laevis*, *Amblyomma variegatum*, *Amblyomma maculatum* and *Ixodes scapularis*. **Figure S3.** The engorged tick weight in dsRNA-SODs injected ticks. The engorged weights of ticks injected with dsLacZ, dsCu/Zn-SOD, dsMn-SOD and dual SODs (dsCu/Zn-SOD and dsMn-SOD) observed at the detachment of ticks from Sheep. There were no significant effects in tick engorged weights with dsRNA-SODs (ANOVA, *F*_(3,52)_ = 0.6274, *P* = 0.6006). (DOCX 473 kb)
