# Supplementary figures and images for: Rickettsia parkeri colonization in Amblyomma maculatum: the role of superoxide dismutases
Source: Parasit Vectors. 2016 May 20;9:291. doi: 10.1186/s13071-016-1579-1 (PMC4873992; doi:10.1186/s13071-016-1579-1)

Fig. S1

Fig. 2SA

**
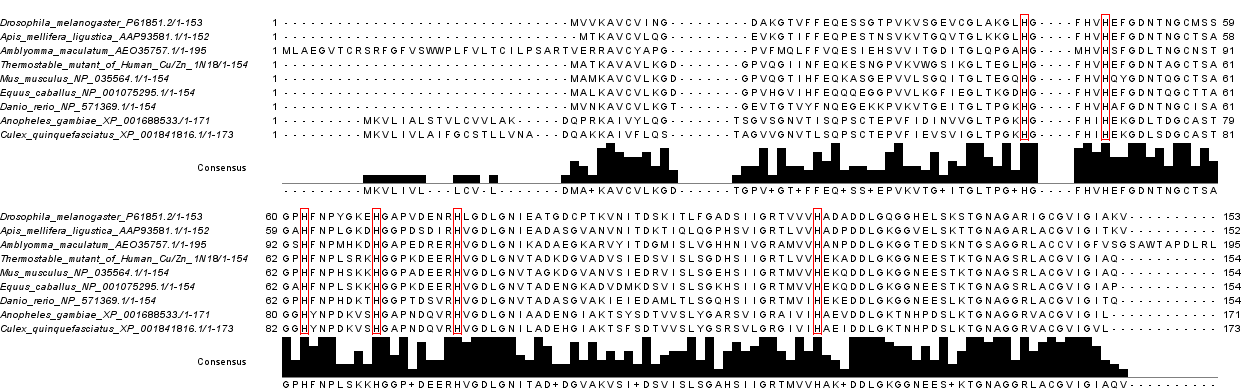
**

Fig 2SB


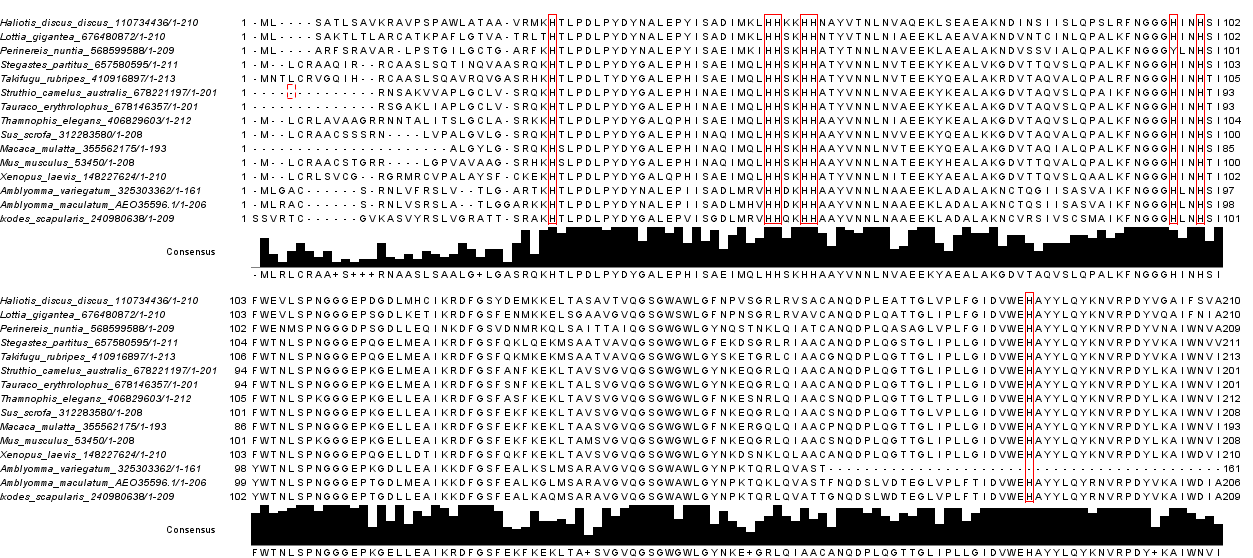


Fig. S3

Supplement: Additional file 1: — Figure S1. Evolutionary relationships of taxa based on the SOD amino acid sequence using maximum likelihood method. The evolutionary history was inferred by using the Maximum Likelihood method based on the JTT matrix-based model [15]. The tree is drawn to scale, with the branch lengths measured by the number of substitutions per site. Evolutionary analyses were conducted using MEGA6 software [14]. The sequences were obtained from Amblyomma maculatum, Amblyomma variegatum, Ixodes scapularis, Anopheles gambiae, Mus musculus, Sus scrofa, and Homo sapiens. GenBank accession numbers followed by species names are shown in the tree. Figure S2A. Multiple sequence alignments of Cu/Zn-SOD amino acid sequences from different taxa. Regions outlined by red boxes indicate metal-binding sites that are conserved between all of the listed species. The sequences for Cu/Zn-SODs were obtained from Drosophila melanogaster, Apis mellifera, Amblyomma maculatum, Thermostable mutant of Human Cu/Zn SOD, Mus musculus, Equus caballus, Danio rerio, Anopheles gambiae and Culex quinquefasciatus. Figure S2B. Multiple sequence alignments of Mn-SOD amino acid sequences from different taxa. Regions outlined by red boxes indicate metal-binding sites that are conserved between all the listed species. The sequences were obtained from Haliotis discus, Lottia gigantea, Perinereis nuntia, Stegastes partitus, Takifugu rubripes, Struthio camelus, Tauraco erythrolophus, Thamnophis elegans, Sus scrofa, Macaca mulatta, Mus musculus, Xenopus laevis, Amblyomma variegatum, Amblyomma maculatum and Ixodes scapularis. Figure S3. The engorged tick weight in dsRNA-SODs injected ticks. The engorged weights of ticks injected with dsLacZ, dsCu/Zn-SOD, dsMn-SOD and dual SODs (dsCu/Zn-SOD and dsMn-SOD) observed at the detachment of ticks from Sheep. There were no significant effects in tick engorged weights with dsRNA-SODs (ANOVA, F (3,52) = 0.6274, P = 0.6006). (DOCX 473 kb) [file 13071_2016_1579_MOESM1_ESM.docx]
